# Bio-Phenolic Resin Derived Porous Carbon Materials for High-Performance Lithium-Ion Capacitor

**DOI:** 10.3390/polym14030575

**Published:** 2022-01-31

**Authors:** Er-Chieh Cho, Cai-Wan Chang-Jian, Cheng-Zhang Lu, Jen-Hsien Huang, Tzu-Hsien Hsieh, Nian-Jheng Wu, Kuen-Chan Lee, Shih-Chieh Hsu, Huei Chu Weng

**Affiliations:** 1 Department of Clinical Pharmacy, School of Pharmacy, College of Pharmacy, Taipei Medical University, 250 Wuxing Street, Taipei City 110, Taiwan; echo@tmu.edu.tw; 2 Department of Mechanical and Automation Engineering, I-Shou University, No. 1, Sec. 1, Syuecheng Rd., Dashu District, Kaohsiung City 84001, Taiwan; cwchangjian@mail.isu.edu.tw; 3 Material and Chemical Research Laboratories, Industrial Technology Research Institute, No. 195, Chung Hsing Road, Chutung, Hsinchu 31040, Taiwan; chengzhanglu@itri.org.tw; 4 Department of Green Material Technology, Green Technology Research Institute, CPC Corporation, No. 2, Zuonan Rd., Nanzi District, Kaohsiung City 81126, Taiwan; 295604@cpc.com.tw (J.-H.H.); d98524001@ntu.edu.tw (T.-H.H.); 5 CNRS, Institut des Sciences Moléculaires d’Orsay, Université Paris-Saclay, 91405 Orsay, France; nianjheng.wu@u-psud.fr; 6 Department of Science Education, National Taipei University of Education, No. 134, Sec. 2, Heping E. Rd., Da-an District, Taipei City 106, Taiwan; 7 College of Medical Science and Technology, Taipei Medical University, Taipei City 110, Taiwan; 8 Department of Chemical and Materials Engineering, Tamkang University, No. 151, Yingzhuan Road, Tamsui District, New Taipei City 25137, Taiwan; 9 Department of Mechanical Engineering, Chung Yuan Christian University, No. 200, Chungpei Road, Chungli District, Taoyuan City 32023, Taiwan

**Keywords:** biomass, porous carbon, Li_4_Ti_5_O_12_, supercapacitor, lithium-ion battery

## Abstract

In this article, hierarchical porous carbon (HPC) with high surface area of 1604.9 m^2^/g is prepared by the pyrolysis of rubberwood sawdust using CaCO_3_ as a hard template. The bio-oil pyrolyzed from the rubber sawdust, followed by the polymerization reaction to form resole phenolic resin, can be used as a carbon source to prepare HPC. The biomass-derived HPC shows a three-dimensionally interconnected morphology which can offer a continuous pathway for ionic transport. The symmetrical supercapacitors based on the as-prepared HPC were tested in 1.0 M tetraethylammonium tetrafluoroborate/propylene carbonate electrolyte. The results of electrochemical analysis show that the HPC-based supercapacitor exhibits a high specific capacitance of 113.3 F/g at 0.5 A/g with superior rate capability and cycling stability up to 5000 cycles. Hybrid lithium-ion capacitors (LICs) based on the HPC and Li_4_Ti_5_O_12_ (LTO) were also fabricated. The LICs have a maximum energy density of 113.3 Wh/kg at a power density of 281 W/kg. Moreover, the LIC also displays a remarkable cycling performance with a retention of 92.8% after 3000 cycles at a large current density of 0.75 A/g, suggesting great potential application in the energy storage of the LIC.

## 1. Introduction

Due to the continuous advancement of science and technology, various electrical appliances and 3C products are widely used by the world. Thus, the world is evolving towards the current situation of high energy consumption. With the continuously increasing demand of energy and serious environmental crisis, the development of clean renewable energy has attracted more attention to reduce dependence on fossil fuels. Unfortunately, the renewable energies such as solar and wind are unstable energy sources altered by climate conditions and cannot provide a stable electric output. Energy storage, which can allow renewable energy to be stored and used later as needed, is emerging as a critical component of clean energy technologies. Therefore, energy storage is becoming increasingly important in order to balance the supply and demand.

Electrical double layer capacitors (EDLC), also known as supercapacitors, are promising for energy storage due to their high-power density, faster charge–discharge rate, better operation safety and much longer cycle life than lithium ion batteries (LIBs). The amount of charge stored in EDLCs depends on the formation of the electric double layer arising between the polarized surface of the working electrode and the ions of the electrolyte. Recently, porous carbon materials have attracted much attention for their applications in EDLCs, owing to their large surface area, diverse pore structure and good chemical and thermal stability. Various carbonaceous materials such as activated carbon [1,2], carbon aerogel [3,4], carbon nanotube [5,6], carbon fiber [7,8,9] and graphene [10,11,12] have been used for the electrode material in EDLCs. Biomass is one of the most abundant renewable resources on earth. It is also an excellent precursor for the production of porous carbon materials from an economic and environmental viewpoint. In a general pyrolysis process, biomass can be converted into bio-oil containing water (15–30 wt%) and more than 100 different organic compounds, including furans, aldehydes, phenols, acids and anhydrosugars [13]. The obtained bio-oil is further processed to produce the HPCs by thermal treatment with physical or chemical activation [14,15]. However, even though a high yield of bio-oil (30–50%) can be obtained from thermal pyrolysis of biomass, its quality is still quite low because of the existence of large amount of water and various small organic molecules [16]. During the carbonization process, the viscous and liquid type of bio-oil is difficult to handle and scale up. In addition, the organic compounds in bio-oil are also easily thermally decomposed leading to a low yield of HPC.

Among the valuable chemicals in bio-oils, they are found to be rich in phenolic compounds with suitable viscosity for the synthesis of resins [17]. To solve the above problem, we developed a modified process to synthesize the HPC from biomass. Here, we further converted the bio-oil derived from rubberwood sawdust into phenolic resins. With the CaCO_3_ as hard templates, the bio-oil-based resol resins are high-quality carbon sources to prepare the HPC via the carbonization process. The solid-state phenolic resins are easily processed with high scalability to produce the HPC. Compared with the raw bio-oil, the HPC prepared with the obtained phenolic resin as the precursor also showed higher yield and could reduce the emission of CO_2_ and volatile organic compounds. The specific surface area of the biomass-derived HPC reaches as high as 1604.9 m^2^/g, which is favorable for the application of EDLC. After coupling the HPC with the high-rate LTO, the hybrid LIC exhibits a high energy/power density of 113.3 Wh/kg at 281 W/kg, and even provided a value of 39.2 Wh/kg at 7031 W/kg.

## 2. Experimental Section

### 2.1. Preparation of HPC

First, rubberwood sawdust was pyrolyzed under a N_2_ atmosphere at 500 °C through a fluidized-bed reactor to produce a bio-oil. Under stirring, the obtained bio-oil was mixed with formaldehyde with a ratio of 4:1 at 50 °C. Subsequently, 50% KOH solution was slowly added into the mixture at 65 °C. After 1 h at 65 °C, the bio-phenols in the bio-oil would participate in a condensation reaction with formaldehyde to form the bio-oil resol resin. The as-prepared bio-oil resin was further blended with the CaCO_3_ template under mechanical stirring. The well-mixed solution was then heated at 80 °C to remove the water. The obtained precursor was then carbonized at 900 °C under a N_2_ atmosphere for 4 h. The product was washed with HCl solution to remove the CaCO_3_ nanoparticles. Finally, the precipitation was washed with deionized water and dried in an oven to give the HPC powder. In this study, four different HPCs were prepared by changing the ratio between bio-oil resin and CaCO_3_ template. We prepared the four samples with CaCO_3_/resin ratios of 0.25, 0.5, 1 and 2, and these samples were denoted as 0.25-HPC, 0.5-HPC, 1-HPC and 2-HPC, respectively. 

### 2.2. Characterization

The crystal structure of the sample was characterized by X-ray powder diffraction (XRD, Philips X’Pert/MPD instrument, El Dorado County, CA, USA). The morphologies were monitored by scanning electron microscopy (SEM, JEOL JSM-6701F, Tokyo, Japan) and transmission electron microscopy (TEM, JEOL 2010, Tokyo, Japan). Thermal gravimetric analysis (TGA) was carried out using a TGA 8000 (PerkinElmer, Boston, MS, USA). Functional group and chemical composition were characterized by using Fourier transform infrared (FTIR, PerkinElmer, Boston, MS, USA) spectroscopy and X-ray photoelectron spectroscopy (XPS, ULVAC-PHI, Tokyo, Japan). Finally, the Raman spectrum of the as-prepared samples was measured by using a Raman microscope (HR800, HORIBA, Tokyo, Japan).

### 2.3. Electrochemical Analysis

HMMC electrodes were prepared by mixing active material (80 wt%), carbon black (15 wt%) and polyacrylic acid (5 wt%) in de-ionized water; LTO electrodes were prepared by mixing the active material, conductive carbon and poly (vinylidene fluoride) at a weight ratio of 80:10:10 in *N*-methylpyrrolidone. The well-mixed slurries were deposited on an aluminum electrode via the doctor-blade method and heated at 120 °C to remove the solvent. The electrodes were cut into a round shape with a diameter of 13 mm and stored under vacuum and elevated temperature (120 °C) to remove the adsorbed moisture. All the electrochemical measurements including cyclic voltammetry (CV) curves, galvanostatic charge/discharge (GCD) profiles and electrochemical impedance spectroscopy (EIS) were performed with an electrochemical workstation (PAR, PARSTAT 4000A, Boston, MS, USA). 

## 3. Results and Discussion

**Figure 1:**
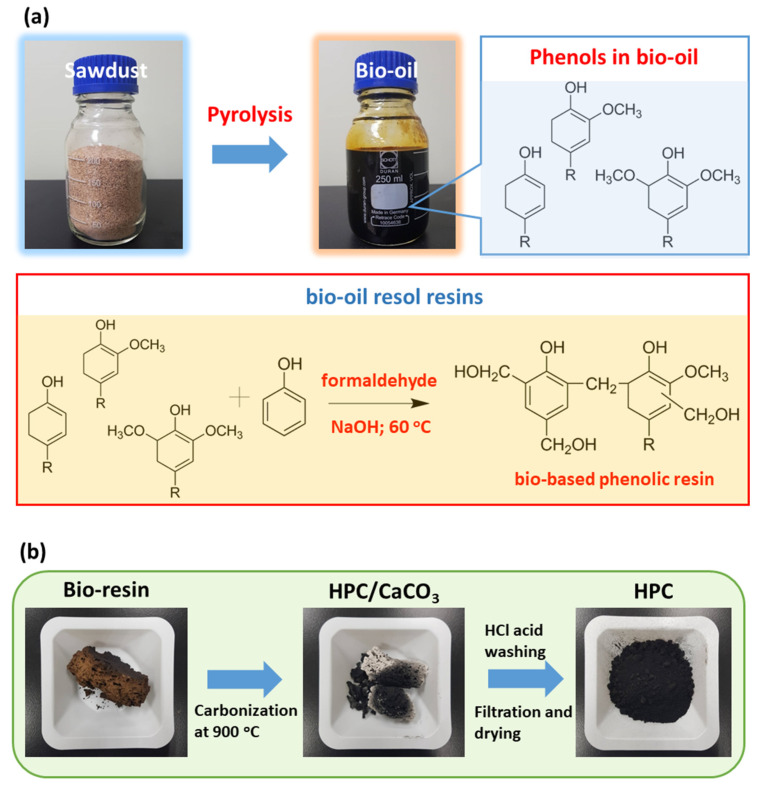
Schematic representation of the resource collection of rubberwood sawdust and its use in the synthesis of the HPC. (**a**) The rubberwood sawdust was pyrolyzed to produce the bio-oil and the bio-phenols in the bio-oil were further reacted with the formaldehyde to obtain bio-based phenolic resin. (**b**) The bio-based resin was further carbonized with CaCO_3_ templates at 900 °C. Afterward, the template was removed by washing with HCl and deionized water. Finally, the biomass-derived HPCs were prepared after drying in an oven.

Figure 1 shows the synthetic route of the HPC using the biomass (rubber wood) as the carbon source in this study. The ground rubber sawdust was pyrolyzed to produce the bio-oil according to our previous report [18]. In general, the resultant bio-oil can be directly used as a carbon source to synthesize the HPC. Unfortunately, the bio-oil contains a large amount of water and various small organic molecules leading to a low yield of the HPC. Here, we propose a modified method to prepare the HPC. We utilize the phenolic compounds in bio-oil to synthesize bio-oil-based resol resins. The resins obtained from the polycondensation reaction between the bio-phenols and formaldehyde are high-quality carbon sources to prepare the HPC with great yield. The obtained resins were further carbonized in the presence of CaCO_3_ template to prepare the HPC. Afterward, the product was washed with HCl solution and deionized water to remove the CaCO_3_ and other impure materials to obtain the HPC.

**Figure 2:**
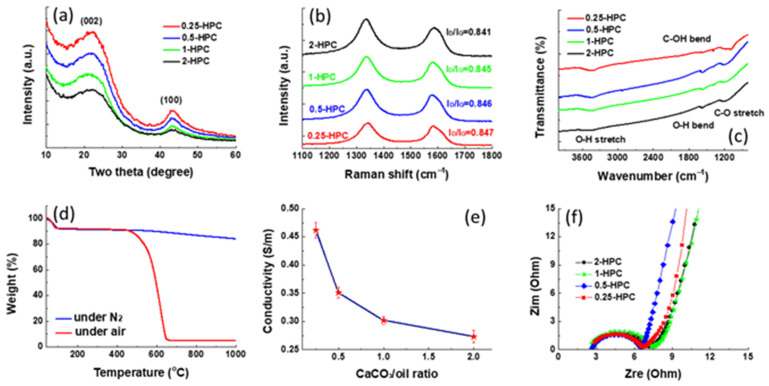
Characterization of the as-prepared HPCs. (**a**) The XRD pattern; (**b**) Raman spectrum; (**c**) FTIR spectrum of the four HPCs; (**d**) the TGA curves of the 2-HPC tested in air and N_2_; (**e**) the conductivity measurement of the four HPCs and (**f**) Nyquist plots of the four HPCs.

Figure 2a shows the XRD pattern of the HPC prepared from rubber wood sawdust at 800 °C with different CaCO_3_/bio-oil ratios. All the samples exhibit two broad diffraction peaks located at 23.2° and 43.3°, which can be assigned to the (002) and (100) planes of graphite like structure [19]. The broad peak indicates the amorphous feature of the carbon material with a disorder structure. With the increase in CaCO_3_ content, it can be seen that the intensity of the diffraction peaks gradually becomes weaker. This illustrates that the CaCO_3_ template can disrupt the graphitic carbon stacking, resulting in more porous carbon materials. The carbon structure was further confirmed by performing the Raman spectroscopy. In Figure 2b, the peaks at 1340 cm^−1^ and 1580 cm^−1^ correspond to the disordered (D-band) defects carbon peak and a graphitic-like crystalline band (G-band), respectively. The graphitization degree depending on the number of defects and disorder structures can be simply evaluated through the intensity ratio of the two peaks (I_D_/I_G_ ratio) [20]. It has been found that the four samples show an almost unchanged I_D_/I_G_ value (0.841–0.847), implying the content of the CaCO_3_ template cannot alter the atomic arrangement and the graphitization degree depends only on the annealing temperature. The surface chemical property of the HPC was investigated by measuring the FTIR spectrum. As shown in Figure 2c, the presence of a broad band between 3600–3300 cm^−1^, represent the O–H stretching vibration [21]. The peaks at 1570 cm^−1^, 1384 cm^−1^ and 1192 cm^−1^ in all samples are attributed to the stretching vibration of C=O [22], C=C aromatics [23] and C–O [24], respectively. The results indicate that the as-prepared PCs still contain some oxygen containing functional groups even after thermal treatment at 800 °C. It has been reported that the moderate amount of oxygen-containing functional groups in carbon materials can improve the wettability of the pore surface and induce additional pseudo-capacitance leading to better capacitive performance [25,26]. To evaluate the content of the oxygen-containing functional groups and ash in the 2-HPC, the TGA analysis was carried out under both air and N_2_ atmospheres. As shown in Figure 2d, the 2-HPC maintained 84% of its weight after heating at 1000 °C under N_2_ atmosphere, suggesting the excellent thermal stability. It can be observed that the 2-HPC lost nearly 5.3% of its weight within 100 °C in air due to the surface adsorbed moisture. Between 470–660 °C, a significant weight loss of 2-HPC can be observed, which originates from the decomposition of carbon–carbon bonds [27]. After heating at 1000 °C under air, the residual content of ash was found to be 4.83%. The electric conductivity (σ) of the four samples is compared in Figure 2e. As the content of CaCO_3_ increased, the σ value of the PC gradually decreased and the mean σ value of the samples was 0.462, 0.351, 0.302 and 0.273 S/cm for 0.25-HPC, 0.5-HPC, 1-HPC and 2-HPC, respectively. It has been reported that the σ strongly depends on the effective electrical contacts established between the sample particles [28]. As expected, the higher content of CaCO_3_ increased the porosity of the carbon materials which reduced the number of channels for the transport of electric current leading to a lower σ value. Moreover, the graphitization degree of the four samples was almost the same (Figure 2b). Therefore, it can be concluded that the σ values of the four HPCs are dominated by their morphologies. Figure 2f shows the Nyquist plot for the different carbon materials. Each Nyquist plot consists of a semicircle at high frequency, followed by a slope line in the low frequency range, which are attributed to charge-transfer impedance and the diffusion of electrolyte ions onto the electrode–electrolyte interface. Although the 0.25-HPC reveals the highest σ value among the four samples, its semicircle in the impedance spectrum is approximately the same size compared with others. This result indicates that the internal resistance of the active layer is dominated by the conductive additive but not by the active material. The above obtained results evidently indicate that the poorly crystallized carbons can be prepared using the rubber wood sawdust as a carbon source.

**Figure 3:**
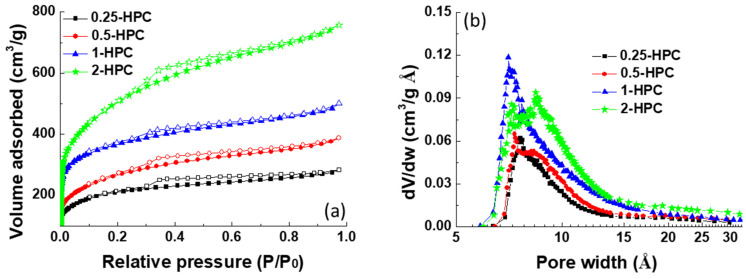
The measurement of the surface area of the HPCs. (**a**) The N_2_ adsorption/desorption isotherms and (**b**) the pore size distribution of the four as-prepared HPCs.

The capacitive performance of carbon materials is directly reflected by the active surface area which determines the specific capacitance and rate capability. Therefore, nitrogen adsorption/desorption isotherms and the corresponding pore size distributions are further studied to explore the pore structure of HPCs with different CaCO_3_ contents, as shown in Figure 3a,b. According to IUPAC classification, the as-synthesized HPCs displayed a type IV isotherm with an associated hysteresis cycle of the H4 type, suggesting the presence of well-developed mesopores in the structure [29]. Among the four samples, the specific surface area of 2-HPC is the largest and the values are calculated to be 1604.9, 1195.5, 855.1 and 685.4 m^2^/g for 2-HPC, 1-HPC, 0.5-HPC and 0.25-HPC, respectively. Moreover, with higher CaCO_3_ content, the pore size distribution of the PC broadened, indicating the formation of a mesoporous structure. These results are in good agreement with the XRD pattern (Figure 2a) and the measurement of conductivity (Figure 2e). The high surface area of 2-HPC can provide more tunnels for ion adsorption, which is favorable for the application of the supercapacitor electrode material. These results clearly demonstrate that the higher content of CaCO_3_ template enhances the development of the porous structure during the thermal process, endowing the resulting carbon materials with pore-size broadened hierarchical pores. The high surface areas and the mesoporous structures of the 2-HPC indicates its great potential for use as an electrode material for energy storage devices.

**Figure 4:**
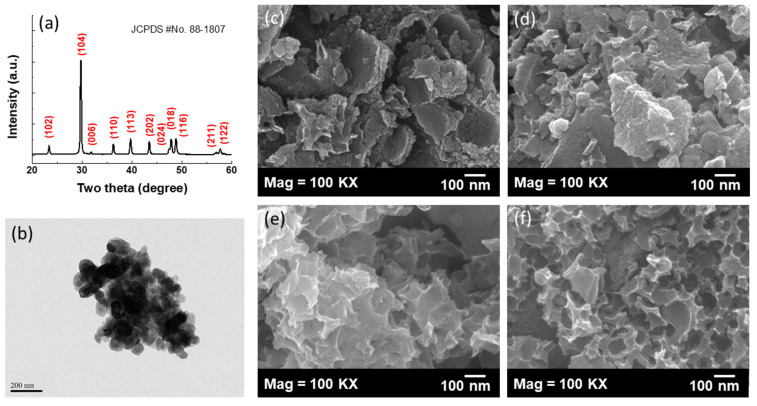
The surface morphology investigation of the HPCs. (**a**) The XRD pattern and (**b**) TEM image of the CaCO_3_ templates; the SEM images of (**c**) 0.25-HPC; (**d**) 0.5-HPC; (**e**) 1-HPC and (**f**) 2-HPC.

Figure 4a,b provides the XRD pattern and TEM image of the CaCO_3_ templates. All the major diffraction peaks at 23.4, 29.8, 31.8, 36.3, 39.8, 43.5, 47.9, 48.9, 56.9 and 57.6° can be indexed to the calcite structure without an impure phase. As shown in Figure 4b, the CaCO_3_ templates reveal a narrow particle size distribution with a mean particle size of around 40 nm. The surface morphology of the HPCs was monitored by SEM observation. With the comparison of the various carbon materials, macropores cannot be observed in both 0.25-HPC and 0.5-HPC as shown in Figure 4c,d. It can be observed that both the 0.25-HPC and 0.5-HPC have various micron-sized particles with an irregular morphology; moreover, no visible pores were observed on the surface of 0.25-HPC and 0.5-HPC. As the content of CaCO_3_ increases, the surface morphology of the products shows a transition from a solid and nonporous structure (0.25-HPC and 0.5-HPC) to a honeycomb structure with abundant interconnected pores (1-HPC and 2-HPC). With a further increase in CaCO_3_ content from 1-HPC to 2-HPC, the surface morphology shows a loosened structure with thinner pore walls. Many roughly spherical nanopores well distributed within or on the surface of the 1-HPC and 2-HPC can be observed in Figure 4e,f, confirming that interconnected pores are created by the hard template of CaCO_3_. Moreover, it also can be observed that these CaCO_3_ templates have been completely removed during the HCl washing process.

**Figure 5:**
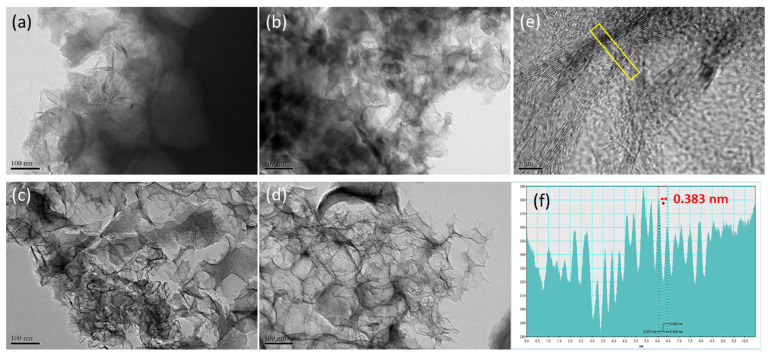
Morphological investigation of the as-prepared HPCs. The TEM morphology of (**a**) 0.25-HPC; (**b**) 0.5-HPC; (**c**) 1-HPC; (**d**) 2-HPC; (**e**) the zoomed in image of 2-HPC and (**f**) contrast profile along the marked region in (**e**) indicate the interlayer distance.

To further investigate the microstructure of the HPCs, the TEM analysis was also explored as shown in Figure 5. All the samples feature an amorphous, graphene-like structure. As shown in Figure 5a, 0.25-HPC shows a thick and solid aggregation without a porous structure. In contrast, 2-HPC presents many three-dimensionally interconnected nanopores uniformly distributed within the carbon materials. With careful observation, the nanopore size (30–80 nm) of 2-HPC is very close to the size of CaCO_3_ nanoparticles, as shown in Figure 4b. In addition, the structure of the pore walls was monitored at higher magnification as shown in Figure 5e. It can be observed that the thickness of the nanopore walls is less than 5 nm with disordered graphite crystalline. The contrast line profile of the yellow-marked region in Figure 5e is also displayed in Figure 5f. Based on the contrast line profile, the interlayer distance of the graphite lattice fringes observed from the pore walls in 2-HPC is around 0.383 nm. The highly porous structure with a localized graphitic structure of 2-HPC is responsible for its high surface area. Taken together, the SEM and TEM images of 2-HPC reveal a highly porous structure having a high specific surface area, both of which would be beneficial to ion storage and ion transport.

**Figure 6:**
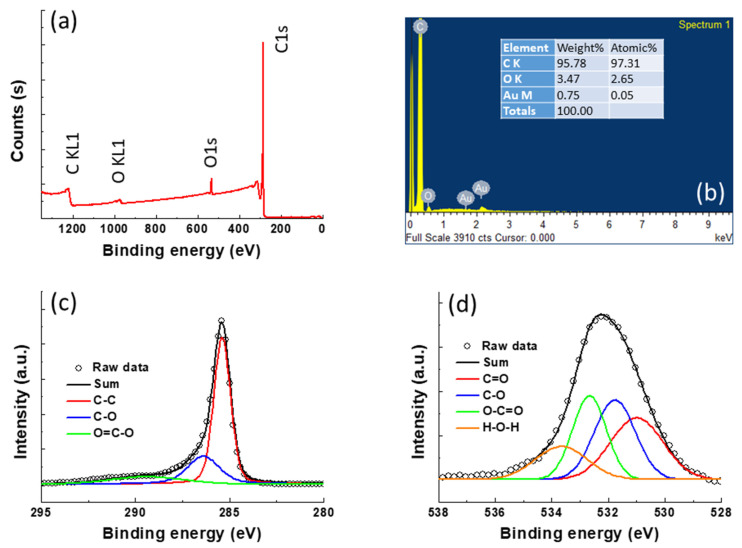
The analysis of chemical composition for the 2-HPC. (**a**) The XPS survey; (**c**) EDS spectrum; deconvolution of the high-resolution XPS spectra of the (**c**) C 1s and (**d**) O 1s binding energies.

The chemical compositions of 2-HPC were further analyzed by XPS and EDS, and the results are shown in Figure 6. As expected, the XPS survey spectrum of 2-HPC as shown in Figure 6a shows the presence of the characteristic C and O elements with energy at around 285 and 533 eV, respectively, indicating the obtained 2-HPC is pure and free from other elements. Moreover, elemental composition of the 2-HPC was further analyzed by EDS as shown in Figure 6b. It can be seen that only C, O and Au can be observed in the 2-HPC and the atomic percentage of C and O is found to be 97.31 and 2.65%, respectively. Here, the Au signal originates from the Au sputtering pre-treatment. Figure 6c exhibits the C1s spectrum, which shows peaks of 285.4, 286.6 and 289 eV, corresponding to C–C, C–O and O=C–O bonds, respectively [30]. The proportion of C–C, C–O and O=C–O can be calculated by the integrated peak area and their values are 62.98, 24.85 and 12.18%, respectively. In addition, the O1s spectrum in Figure 6d also exhibits four main peaks with a binding energy at about at 531, 531.7, 533 and 534.7 eV, representing C=O, C–O, O–C=O and adsorbed H_2_O molecules [31] whose proportions are 29.47, 29.28, 25.46 and 15.79%, respectively. It has been observed that these oxygen-containing functional groups can positively affect the integral capacitance, due to the additional pseudocapacitance provided by the oxygen functional groups [25,26].

**Figure 7:**
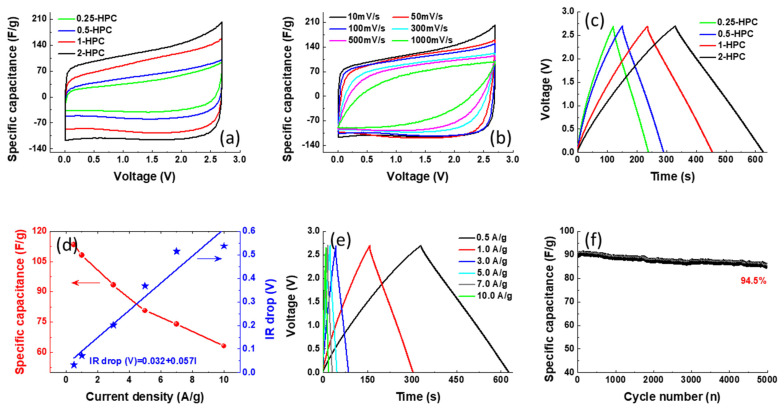
Electrochemical characterization of the as-prepared HPC. (**a**) The CV curves of the four different HPCs stepped between 0 and 2.7 V with a scan rate of 10 mV/s; (**b**) CV curves of the 2-HPC, recorded at various scan rates; (**c**) GCD profiles of the four HPCs, recorded at 0.5 A/g; (**d**) the rate performance and the IR drop of 2-HPC; (**e**) the corresponding GCD profiles of the 2-HPC; (**f**) cycling performance of the 2HPC-based device, tested at a current of 3 A/g.

The capacitive behavior of the as-synthesized HPCs was characterized using CV and GCD measurements in a two-electrode symmetric cell configuration. Figure 7a shows the CV curve of the HPCs from 0 to 2.7 V at a scan rate of 10 mV/s. All samples reveal the typical EDLC behavior with a quasi-rectangular CV shape, indicating the excellent electrochemical property. Owing to its unique porous architecture with three-dimensionally interconnected morphology as confirmed by the SEM and TEM observation, the 2-HPC displays the largest CV loop area, implying its high specific capacitance. Figure 7b shows the CV curves of 2-HPC within scan rates of 10~1000 mV/s. With an increasing scan rate, the plateau current increases accordingly and the CV curve can be roughly maintained, suggesting a good rate capability due to the low inner resistance and fast electrolyte ion diffusion kinetics. The GCD curves of the HPCs obtained at a current density of 0.5 A/g are presented in Figure 7c. All the GCD curves are highly linear and symmetric, suggesting the electrochemical reversibility of 2-HPC. The specific capacitances from the GCD plots were calculated using Equation (1):

(1) $$C=2\cdot \frac{I\cdot \Delta t}{m\cdot \Delta V}\;\;$$

where, *I*, Δ*t*, *m* and Δ*V* represent the discharging current, discharge time, mass of active material in one electrode and potential window, respectively. The specific capacitance of the carbon materials is calculated to be 41.6, 51.9, 81.1 and 113.3 F/g for 0.25-HPC, 0.5-HPC, 1-HPC and 2-HPC, respectively. The specific capacitance and IR drop as a function of current density for 2-HPC are shown in Figure 7d. As the current density increases, the specific capacitances of 2-HPC gradually decrease and the capacitance is 63 F/g with a capacitance retention of 55.6% at 10 A/g. The corresponding charge-discharge profiles with different current densities of 2-HPC are also exhibited in Figure 7e. The IR drop is attributed to the equivalent series resistance of the device, which is obtained from the instantaneous voltage drop at the beginning of the discharging state. The increase in resistance with respect to applied current densities is almost linear (IR drop [V] = 0.032 + 0.057I). The small IR drop values facilitate high discharge power delivery in practical applications, which is attributable to the hierarchically porous and interconnected structure of 2-HPC. Figure 7f presents the cycling performance of a 2-HPC-based supercapacitor tested at a current density of 3 A/g. It shows a good cycling stability and retains the initial discharge capacitance to the extent of 94.5% after 5000 cycles, indicating the interfacial stability for an active material-self and active material-current collector [32]. The properties of various biomass-based porous carbons and 2-HPC prepared in this study are summarized in Table 1. It can be observed that the specific capacitance obtained from the 2-HPC can rival those of the reported values delivered by other biomass-derived porous carbons. The great electrochemical performance of 2-HPC can be attributed to its favorable morphology and high surface area.

**Figure 8:**
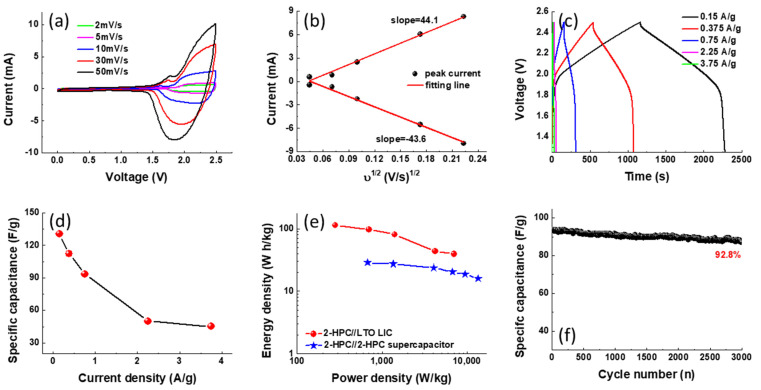
Fabrication of 2-HPC//LTO LICs and their capacitive performance. (**a**) CV curves of the LIC, recorded at various scan rates; (**b**) Linear relationships between the anodic and cathodic peak currents of the LIC and the square root of the scan rate (υ^1/2^); (**c**) the GCD profiles of the LIC, recorded at various currents; (**d**) the rate performances of the 2-HPC//LTO LIC; (**e**) Ragone plots of the 2-HPC//2-HPC and 2-HPC//LTO systems and (**f**) long-term cycling performance of the 2-HPC//LTO LIC tested at 0.75 A/g.

To further explore the practical application of 2-HPC, we also fabricated LICs based on 2-HPC and LTO as cathode and anode, respectively. The LTO materials were prepared according to our previous report [46]. As we all know, in this configuration, the hybrid LICs can combine the advantages of both a lithium ion battery and a supercapacitor and exhibit relatively high energy and power densities [47,48]. Figure 8a shows the CV curves of the LIC between 0 and 2.5 V with different scan rates. It can be seen that the charging process is activated at a voltage higher than 1.5 V, because the LTO could store charge through the insertion of Li^+^ ions only at voltages of greater than 1.5 V [49]. The broad anodic and reduction peaks observed at around 2.2 and 1.9 V result from the redox reaction of Li^4+^/Li^7+^ couples [50]. Figure 8b shows the plot of peak current (*i*) versus the square root of the scan rate (υ^1/2^) for the as-fabricated LiC. The linear relationship indicates that the redox reaction is dominated by a diffusion-controlled process. Moreover, the slops of the anodic and cathodic process in the *i*-υ^1/2^ plot are very close, suggesting excellent electrochemical reversibility and capacitive characteristics. The GCD profiles of the LIC recorded at different current densities are shown in Figure 8c to investigate its energy storage performance. According to the GCD profile, we calculate the specific capacitance based on Equation (2):

(2) $$C=\frac{I\cdot \Delta t}{\left(m_{a}+m_{b}\right)\cdot \Delta V}\;\;\;$$

where *m_a_* and *m_c_* are the weight of the active material for the anodic and cathodic electrodes, respectively. As shown in Figure 8d, the specific capacitance calculated based on the total mass of the two electrodes achieves 130.5 F/g at a current density of 0.15 A/g, and the device shows a retention of 34.6% (45.2 F/g) at 3.75 A/g. Figure 8e shows the Ragone plots to compare the energy and power property of the 2-HPC//2-HPC symmetric supercapacitor and 2-HPC//LTO hybrid LIC. Obviously, the overall energy density of 2-HPC//LTO is higher than that of a 2-HPC//2-HPC symmetric device. The hybrid LIC exhibits promising energy density of 113.3 and 39.2 W h/kg at a power density of 281 and 7031 W/kg, respectively. The cycling stability was estimated by repeating GCD measurements at 0.75 A/g for 3000 cycles. The final specific capacitance still retains 92.8% of its original value (87.3 F/g), indicating its excellent cycling stability. With the combined advantages of the 2-HPC and LTO, the resulting hybrid LIC demonstrates remarkable power and energy densities and an excellent cycle life.

## 4. Conclusions

In summary, we have applied the resole phenolic resin derived from biomass as the carbon source to produce hierarchically porous carbon materials. The resultant 2-HPC exhibits superior capacitive performances on a symmetric supercapacitor in the aspects of high specific capacitance (113.3 F/g at 0.5 A/g) and ultralong cycle stability (94.5% capacitance retention after 5000 cycles at 3 A/g). The excellent performance is attributed to its high surface area and unique three-dimensionally interconnected morphology that is favorable for the transport of ions and electrical contact. The hybrid LIC combined with 2-HPC and LTO also displays an outstanding high energy density of 39.2 W h/kg even at a high-power density of 7031 W/kg. The capacity retention is up to 92.8% after 3000 ultra-long cycles, corresponding to 0.0024% fading per cycle. This work provides an efficient approach to covert the biomass waste into porous carbon materials satisfying the requirements of energy storage devices. For the future investigation, the nitrogen-doped 2-HPC can be prepared using melamine as a nitrogen source to further improve its electrochemical properties. The nitrogen-doped 2-HPC can be integrated with TiNb_2_O_7_ which is a cathode material with higher theoretical capacity than that of LTO to fabricate a novel LIC. With the combination of nitrogen-doped 2-HPC and TiNb_2_O_7_, it is expected that both the power and energy densities of the doped 2-HPC//TiNb_2_O_7_ LIC can be enhanced compared with that of 2-HPC//LTO LIC.

## Figures and Tables

**Table 1:** Comparison of different biomass-based porous carbons (from the literature) with the 2-HPC prepared in this study.

Biomass Resource	BET Surface Area (m^2^/g)	Capacitance (F/g)	Measurement Condition	Ref
Corn cob	567.36	118	0.5 A/g (6 M KOH)	[33]
Industrial waste cotton	1893	118	0.5 A/g (1 M TEABF_4_/AN)	[34]
Cotton	1284	92	0.1 A/g (6 M KOH)	[35]
Lignin	2170	87		[36]
Quinoa	2597	99.2	0.5 A/g (1 M TEABF_4_/AN)	[37]
European hornbeam	614	24	0.25 A/g (1.0 M H_2_SO_4_)	[38]
Rice husk	1984	42.5	0.5 A/g (6 M KOH)	[39]
Miscanthus grass	2062	162	0.5 A/g (6 M KOH)	[40]
Pinecone	1169.31	43	0.5 A/g (1 M H_2_SO_4_)	[41]
Chrysopogon zizanioides		120	0.2 A/g (6 M KOH)	[42]
Coir pith bio-waste	453	33.7	0.2 A/g (6 M KOH)	[43]
Chinese parasol fluff	1449	33.8	0.5 A/g (BMIMBF_4_/AN)	[44]
Areca palm leaves	876	132	0.5 A/g (PVA–Li_2_SO_4_)	[45]
Rubberwood sawdust	1604.9	113.3	0.5 A/g ((C_2_H_5_)_4_N(BF_4_)/PC)	This work

## Data Availability

Not applicable.
